# Nox1 Oxidase Suppresses Influenza A Virus-Induced Lung Inflammation and Oxidative Stress

**DOI:** 10.1371/journal.pone.0060792

**Published:** 2013-04-08

**Authors:** Stavros Selemidis, Huei Jiunn Seow, Brad R. S. Broughton, Antony Vinh, Steven Bozinovski, Christopher G. Sobey, Grant R. Drummond, Ross Vlahos

**Affiliations:** 1 Department of Pharmacology, Monash University, Clayton, Victoria, Australia; 2 Department of Pharmacology, University of Melbourne, Parkville, Victoria, Australia; Albany Medical College, United States of America

## Abstract

Influenza A virus infection is an ongoing clinical problem and thus, there is an urgent need to understand the mechanisms that regulate the lung inflammation in order to unravel novel generic pharmacological strategies. Evidence indicates that the Nox2-containing NADPH oxidase enzyme promotes influenza A virus-induced lung oxidative stress, inflammation and dysfunction *via* ROS generation. In addition, lung epithelial and endothelial cells express the Nox1 isoform of NADPH oxidase, placing this enzyme at key sites to regulate influenza A virus-induced lung inflammation. The aim of this study was to investigate whether Nox1 oxidase regulates the inflammatory response and the oxidative stress to influenza infection *in vivo* in mice. Male WT and Nox1-deficient (Nox1^−/y^) mice were infected with the moderately pathogenic HkX-31 (H3N2, 1×10^4^ PFU) influenza A virus for analysis of bodyweight, airways inflammation, oxidative stress, viral titre, lung histopathology, and cytokine/chemokine expression at 3 and 7 days post infection. HkX-31 virus infection of Nox1^−/y^ mice resulted in significantly greater: loss of bodyweight (Day 3); BALF neutrophilia, peri-bronchial, peri-vascular and alveolar inflammation; Nox2-dependent inflammatory cell ROS production and peri-bronchial, epithelial and endothelial oxidative stress. The expression of pro-inflammatory cytokines including CCL2, CCL3, CXCL2, IL-1β, IL-6, GM-CSF and TNF-α was higher in Nox1^−/y^ lungs compared to WT mice at Day 3, however, the expression of CCL2, CCL3, CXCL2, IFN-γ and the anti-inflammatory cytokine IL-10 were lower in lungs of Nox1^−/y^ mice vs. WT mice at Day 7. Lung viral titre, and airways infiltration of active CD8^+^ and CD4^+^ T lymphocytes, and of Tregs were similar between WT and Nox1^−/y^ mice. In conclusion, Nox1 oxidase suppresses influenza A virus induced lung inflammation and oxidative stress in mice particularly at the early phases of the infection. Nox1 and Nox2 oxidases appear to have opposing roles in the regulation of inflammation caused by influenza A viruses.

## Introduction

Influenza A virus infections represent important infectious diseases that continue to inflict significant global morbidity and mortality [1]. The effects of influenza infection vary from strain to strain, ranging from transient debilitating respiratory illness to more severe respiratory complications that are sometimes fatal. Seasonal and pandemic influenza infections over the last century have claimed over 50 million lives and impose a huge socio-economic burden [2].

Accumulated animal and human studies provide compelling evidence for a new paradigm whereby excessive production of reactive oxygen species (ROS), such as superoxide anion, and its derivatives hydrogen peroxide and peroxynitrite, are crucial mediators of the acute lung injury to influenza A virus infection [3]–[5]. However, although the evidence supporting a pathogenic role for ROS in lung injury to influenza A virus is strong, little attention has been directed towards identifying the key enzymes that generate ROS. Knowledge of the culprit enzymes could give rise to novel pharmacological strategies for manipulating oxidative stress and the associated lung injury following influenza A virus infection.

Recently the Nox2 isoform of the NADPH oxidase family of superoxide-generating enzymes was identified as a major player in the lung pathology caused by influenza A virus infection. Mice genetically deficient in the Nox2 subunit or in a key regulatory subunit of Nox2 activity, p47phox, demonstrated substantially lower: 1) superoxide production by bronchoalveolar lavage fluid (BALF) inflammatory cells and lung oxidative stress; 2) lung oedema and injury; 3) alveolar lung epithelial apoptosis; and 4) peribronchial inflammation compared to WT mice [4], [6], [7]. Moreover, a lack of Nox2 oxidase activity resulted in improved lung function [8]. Despite the reduction in airways and BALF inflammation, viral clearance was not compromised but was significantly improved in the Nox2 deficient mice [6], [8]. Finally, the protective effects of Nox2 deficiency against influenza A virus infection appeared to occur irrespective of the infecting strain, highlighting the exciting therapeutic potential of targeting Nox2 oxidase [6], [7].

Notwithstanding the important role of Nox2, a number of additional sources of superoxide, such as the Nox1 isoform of the NADPH oxidase family, are expressed in lungs and may therefore influence the inflammatory response to influenza A virus infection. It is noteworthy that Nox1 mRNA has been identified in lung epithelial and endothelial cells, potentially placing the enzyme at key sites to regulate cytokine production and lung inflammation following an influenza viral infection [9]. However, it is so far unknown if Nox1 influences lung inflammation in response to influenza A virus infection. Thus, in the present study we performed an extensive phenotypic analysis of the clinical features of influenza A virus infection in the novel Nox1-deficient mouse. The present study shows that Nox1 oxidase critically inhibits the early burst in lung pro-inflammatory cytokine expression, inflammation and oxidative stress caused by influenza A virus infection and therefore, as opposed to the Nox2 oxidase, Nox1 is a protective mechanism against such infections.

## Results

### Expression of Nox1

**Figure 1:**
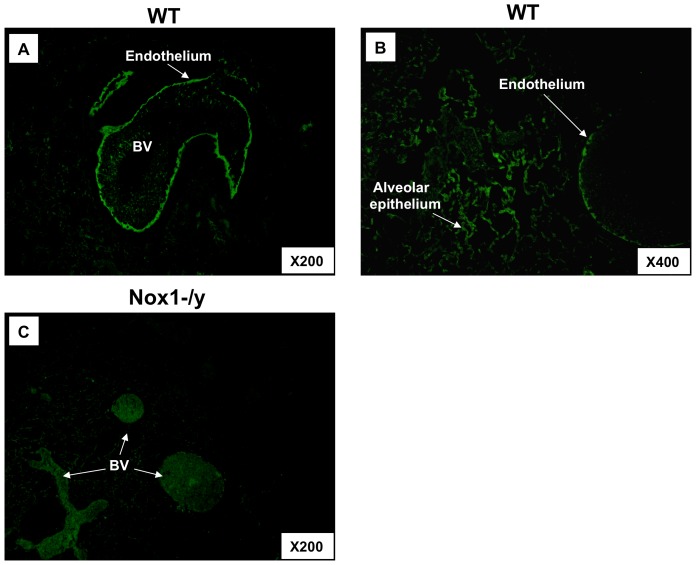
Localization of Nox1 to lung endothelium and alveolar epithelial cells in WT and Nox1^−/y^ mice. Representative photomicrographs (n = 5) show cellular localization of Nox1 in lungs from WT (A–B) and Nox1^−/y^ (C) mice infected with HkX-31 (H3N2) influenza A virus on Day 3. Note, magnification of X200 and X400. Nox1 immunoreactivity is shown in green. BV = blood vessel.

A Nox1 specific antibody revealed strong immunofluorescence in the endothelium of blood vessels and in alveolar epithelial cells of lung sections taken from WT mice that was not evident in equivalent lung sections from Nox1^−/y^ mice (Figure 1). The lack of fluorescence in the Nox1^−/y^ lung sections verifies the specificity of the antibody for Nox1.

### Pro-inflammatory Cytokine and Chemokine Expression in Lungs from Nox1^−/y^ Mice

**Figure 2:**
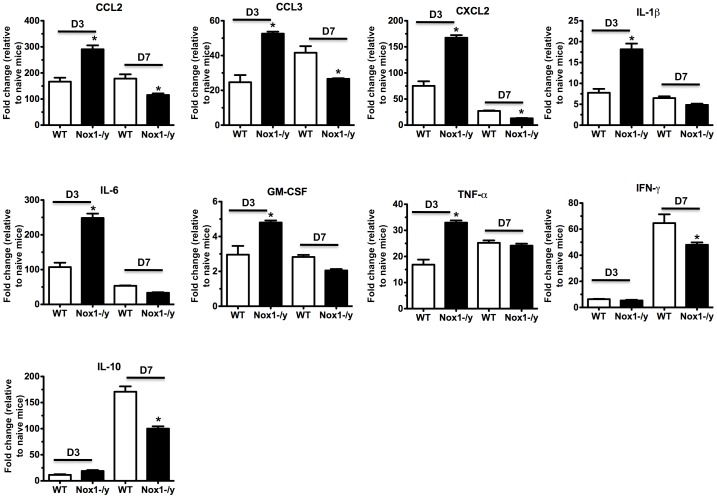
Lung cytokine and chemokine levels. Effect of HkX-31 (H3N2) influenza A virus infection on CCL2, CCL3, CXCL2, IL-1β, IL-6, TNF-α, IFN-γ, GMCSF and IL-10 mRNA expression in whole lung obtained from WT and Nox1^−/y^ mice 3 days and 7 days post infection. Gene expression levels are shown as fold change relative to naïve (uninfected) WT mice shown in supplementary Figure E1 after normalisation to 18S rRNA (housekeeping gene). Data are shown as mean ± SEM of 4 individual mice. *P<0.05 vs WT (HkX-31) (Student’s unpaired *t* test).

We assessed mRNA levels of several pro-inflammatory and anti-inflammatory (IL-10) cytokines and chemokines in the lungs of naïve and HkX-31 infected WT and Nox1^−/y^ mice at early (i.e. Day 3) and later (i.e. Day 7) stages of the infection phase with quantitative real time PCR. Naïve WT and Nox1^−/y^ mice had similar levels of CCL3, CXCL2, IL-β, IL-6, TNF-α, IFN-γ, GM-CSF and IL-10 although CCL2 levels were ∼1.7 fold higher in the Nox1^−/y^ lungs (Figure S1). At 3 days post HkX-31 infection in WT mice the levels of all the cytokines and chemokines examined were significantly elevated (Figure 2). However, the levels of all the cytokines and chemokines examined, with the exception of IFN-γ and IL-10 were elevated in Nox1^−/y^ mice following influenza infection and to a greater extent (≥2–3 fold) than in WT mice (Figure 2). By contrast, at 7 days post infection, the levels of CCL2, CCL3, CXCL2, IFN-γ and the anti-inflammatory cytokine IL-10 were significantly lower in influenza A virus-infected Nox1^−/y^ lung compared to WT control, whereas IL-β, IL-6, TNF-α, and GM-CSF were similar between the two strains of mice (Figure 2).

### Histological Investigation of Lung Inflammation

**Figure 3:**
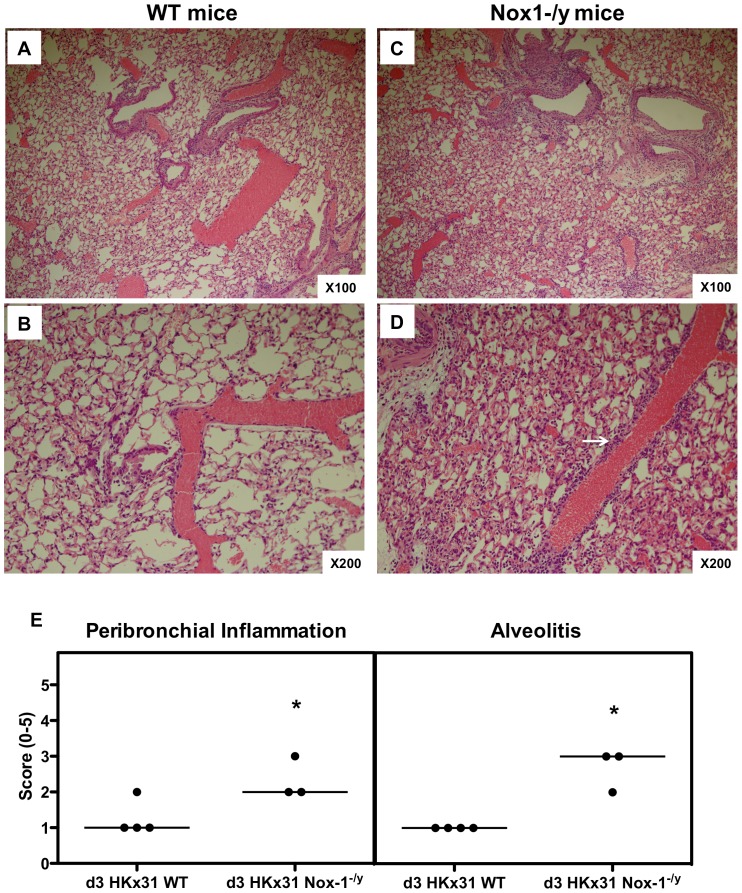
Histological investigation of lung inflammation in HkX-31 virus-infected WT and Nox-1^−/y^ mice 3 days post infection. (A–D) Hematoxylin and eosin stained paraffin sections of lungs from WT and Nox1^−/y^ mice obtained at Day 3. WT mice lungs had obvious peribronchial and perivascular inflammation. However, Nox1^−/y^ mice displayed considerably more peribronchial and perivascular inflammation (shown in bold white arrow) and mild degrees of alveolitis. Note, magnification of ×100 and ×200. (E) Corresponding histopathological scores for peribronchial inflammation and alveolitis. Lung sections were scored blind for alveolitis and peribronchiol inflammation from 0 to 5 as described in *Materials and Methods*. Data shown represent scores from individual mice (as indicated by circles) and median values (as indicated by bar) obtained from one of two independent readers. For each reader, peribronchial inflammation and alveolitis were significantly higher in HKx31-infected Nox-1^−/y^ mice compared with HkX-31-infected WT mice (*P<0.05, Mann-Whitney Wilcoxon test).

**Figure 4:**
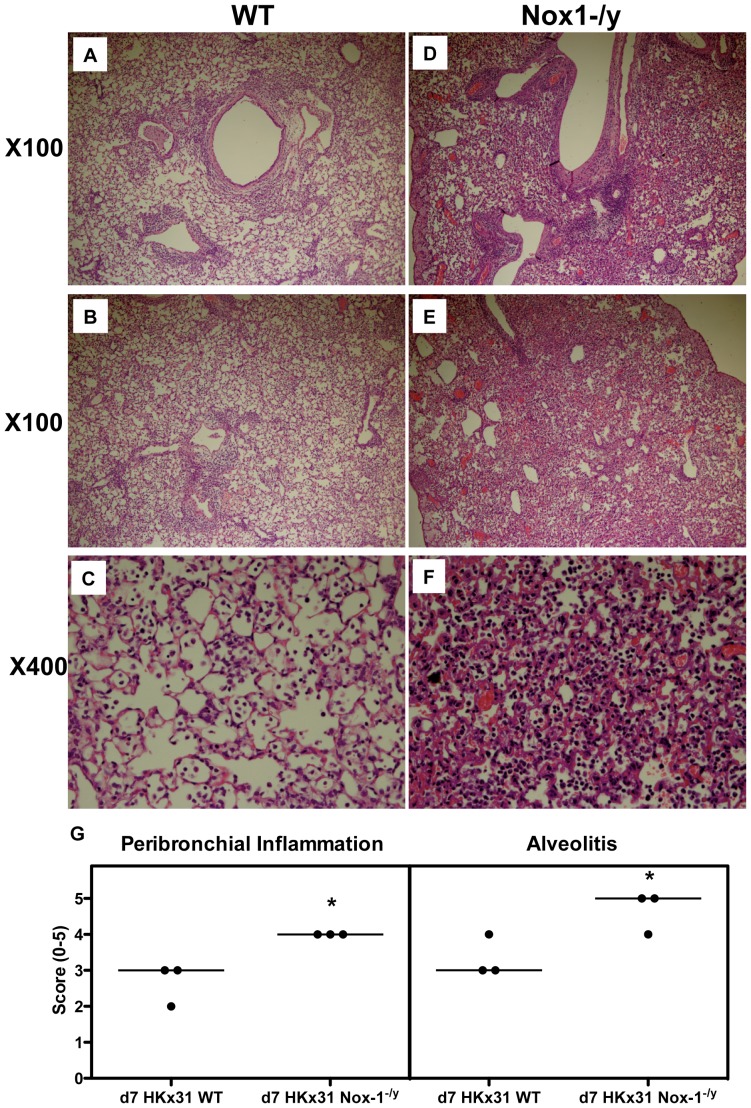
Histological investigation of lung inflammation in HkX-31 virus-infected WT and Nox-1^−/y^ mice 7 days post infection. Hematoxylin and eosin stained paraffin sections of lungs from WT and Nox1^−/y^ mice obtained at Day 7. WT mice lungs had some peribronchial and perivascular inflammation (A–C). However, Nox1^−/y^ mice displayed markedly greater degrees of peribronchial and perivascular inflammation with higher levels of alveolitis (D–F). Note, magnification of ×100 and ×400. (G) Corresponding histopathological scores for peribronchial inflammation and alveolitis. Lung sections were scored blind for alveolitis and peribronchial inflammation from 0 to 5 as described in *Materials and Methods*. Data shown represent scores from individual mice (as indicated by circles) and median values (as indicated by bar) obtained from one of two independent readers. For each reader, peribronchial inflammation and alveolitis were significantly higher in HkX-31-infected Nox-1^−/y^ mice compared with HKx31-infected WT mice (*P<0.05, Mann-Whitney Wilcoxon test).

At 3 days post infection, there was evidence of peri-bronchial inflammation, inflammatory cell exudates in some airways and signs of peri-vascular inflammation in the lungs of WT mice (Figure 3). However, the degree of peri-bronchial and peri-vascular inflammation was substantially greater in the lungs of Nox1^−/y^ mice with evidence of alveolitis (Figure 3 and Figure S2). At Day 7 post infection, WT mice demonstrated a greater degree of airways inflammation compared to Day 3 characterized by bronchial thickening, peri-bronchial and peri-vascular inflammation and a mild degree of alveolitis (Figure 4). The overall peri-bronchial, and in particular, the peri-vascular inflammation observed in the Nox1^−/y^ lung sections was substantially greater (Figure 4). Also, there was evidence of significant and more widespread alveolitis (Figure 4).

### Airway Cellularity in Response to Hk-X31

**Figure 5:**
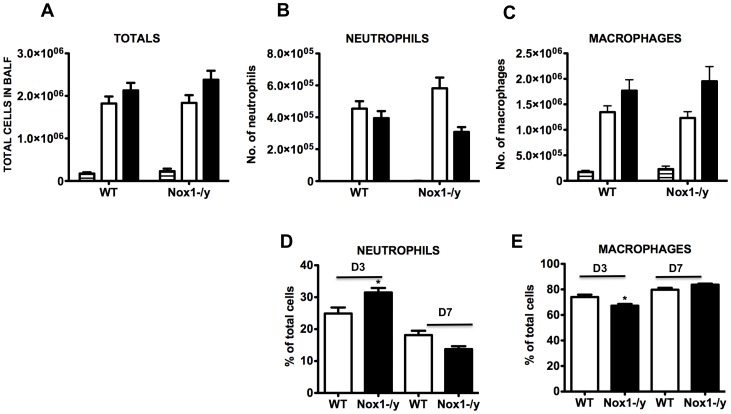
Effect of HkX-31 influenza A virus infection on BALF cellularity in WT and Nox1^−/y^ mice. Mice were treated with 1×10^4^ PFU of HkX-31 (H3N2) strain of influenza A virus and the number of (A) total cells, (B) neutrophils and (C) macrophages, and the percentage of (D) neutrophils and (E) macrophages counted in cytospin preparations of BALF 3 and 7 days post infection. In (A–C) Naïve (horizontal hash), HkX-31 (Day 3; open histogram) and HkX-31 (Day 7; filled histogram). Data are shown as mean ± SEM for 7–12 mice per group. *P<0.05 vs WT (ANOVA and Dunnett’s *post hoc* test).

The extent of cellular infiltration in the BALF of WT and Nox1^−/y^ mice infected with HkX-31 virus was investigated at 3 and 7 days post infection. Naïve Nox1^−/y^ mice had similar total cell numbers in the BALF to naïve WT mice (Figure 5). However, 3 days after infection, the cellular infiltrate in WT mice increased by ∼10 fold from basal levels, which was similar in Nox1^−/y^ mice (Figure 5). By Day 7 post infection, the total number of cells in Nox1^−/y^ mice was similar again when compared to WT mice (Figure 5). Analyses of the infiltrating and resident populations revealed that at 3 days post HkX-31 infection, BALF from Nox1^−/y^ mice contained a strong trend for higher numbers of neutrophils than WT mice (Figure 5). When these data were expressed as a percentage of the total number of cells in the BALF, the proportion of neutrophils from Nox1^−/y^ mice was significantly higher (*P*<0.05) than WT mice (Figure 5). Analysis of macrophages indicated no significant difference in the total numbers of the cells between the two strains of mice, however, the percentage of macrophages was modestly but significantly lower in Nox1^−/y^ mice than WT mice (Figure 5). By day 7, there were no significant differences in total numbers or percentages of neutrophils or macrophages in the BALF between the WT and Nox1^−/y^ mice (Figure 5).

### Effect of Nox1 Deletion on Nox2-dependent Superoxide Production in BALF Inflammatory Cells

**Figure 6:**
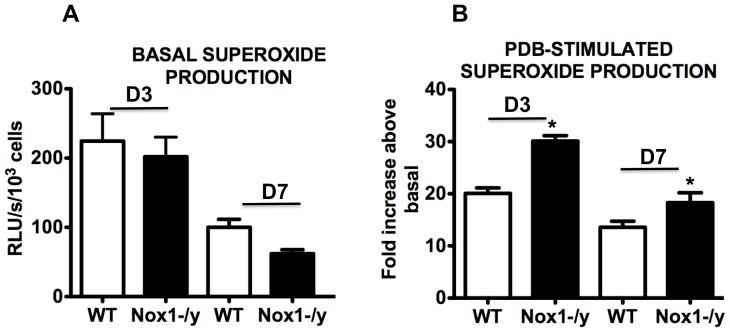
Superoxide production from BALF cells. BALF cells were obtained from HkX-31 influenza A virus-infected WT and Nox1^−/y^ mice 3 days post infection and (A) basal and (B) phorbol dibutyrate (1 µM)-stimulated superoxide production was measured by L-O12 (5 µM) enhanced chemiluminescence. Note, in (B) the PDB-dependent superoxide production was represented as a fold increase above basal levels, which is a measure of the oxidative burst capacity of the cells. Data are shown as mean ± SEM for 7–12 mice per treatment group. *P<0.05 vs WT (ANOVA and Dunnett’s *post hoc* test).

We examined basal and phorbol dibutyrate (PDB)-stimulated superoxide production (oxidative burst solely due to Nox2 oxidase [6]) by inflammatory cells in BALF of HkX-31-infected WT and Nox1^−/y^ mice. BALF inflammatory cells from WT mice infected with HkX-31 produced superoxide under basal conditions and in the presence of PDB, superoxide production increased by ∼20 fold (Figure 6). BALF inflammatory cells from Nox1^−/y^ produced similar levels of basal superoxide to WT mice, however, the PDB-stimulated signal was significantly higher (∼30 fold higher than basal) than that observed in WT mice (Figure 6). The increase in PDB-dependent superoxide generation was also significantly greater in the Nox1^−/y^ BALF cells compared to WT cells at Day 7 (Figure 6).

We assessed whether there was a compensatory alteration in Nox2 expression in the lungs of Nox1^−/y^ mice that could explain the increase in PDB-dependent superoxide production in the BALF inflammatory cells of Nox1^−/y^ mice. Using Western blotting, there were no differences observed in the expression of Nox2 in naïve Nox1^−/y^ mice lungs and in Nox1^−/y^ lungs at D3 and D7 post infection compared to WT mice (Figure S3).

### Effect of Nox1 Deletion on Lung Peroxynitrite Production and Localization

**Figure 7:**
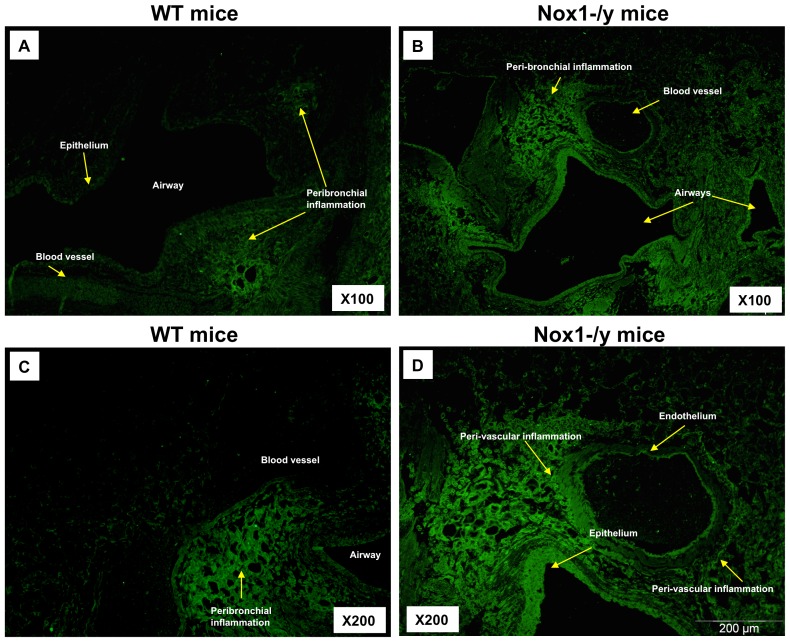
Effect of HkX-31 influenza A virus infection on lung oxidative stress (i.e. peroxynitrite levels). Representative sections of lung tissue obtained from WT (A & C) and Nox1^−/y^ mice (B & D) infected with HkX-31 were incubated with mouse monoclonal anti-3-nitrotyrosine antibody (1∶50) followed by biotinylated anti-mouse IgG reagent. WT mouse lung sections displayed immunofluorescence for 3-NT in the peribronchial regions, in inflammatory cells that infiltrated the airways and in the alveolar tissue and in some regions of the epithelial and endothelial cell layer. In contrast, Nox1^−/y^ mice displayed markedly greater degrees of immunofluorescence for 3-NT and distribution. In particular there was substantially greater degrees of immunofluorescence in the peri-bronchial and peri-vascular regions (mainly due to inflammatory cells and alveolar epithelial cells), endothelial cells and in epithelial cells of most airways examined compared to the WT mice.

Lung sections taken at Day 3 from WT mice infected with HkX-31 displayed immunofluorescence for 3-nitrotyrosine in the inflammatory cells that infiltrated the airways and in peri-bronchial regions (Figure 7). 3-NT staining was also visible in the alveolar space, associated with inflammatory cells (Figure 7). Of note, very little peri-vascular 3-NT staining was observed in the blood vessels surrounding the airways. By contrast, the overall 3-NT immunofluorescence intensity observed in lung sections obtained from Nox1^−/y^ mice infected with HkX-31 was markedly greater (Figure 7) particularly in the peri-bronchial and peri-vascular regions, as well as the epithelium. Strikingly, nearly all of the airways observed had significant fluorescence in the epithelium. Also evident was a significant degree of 3-NT fluorescence in the endothelium of blood vessels that were in close proximity to major airways (Figure 7).

At Day 7 post infection, 3-NT fluorescence was still evident in the lung sections of WT mice with a similar pattern of fluorescence as seen at Day 3 including peri-bronchial and epithelial fluorescence (Figure S4). The intensity of 3-NT staining in lungs from Nox1^−/y^ mice was again markedly greater with nearly all epithelial cell layers of the airways observed to possess significant 3-NT fluorescence.

We also performed 3-NT immunofluorescence in lung sections taken from HkX-31-infected Nox2^−/y^ mice. There was a clear reduction in the 3-NT fluorescence intensity in the airways epithelium, peri-bronchial regions and endothelium of blood vessels in sections from Nox2^−/y^ vs WT mice (Figure S5 and [6]).

### Effect of Nox1 Deletion on Bodyweight Loss and Viral Titers

**Figure 8:**
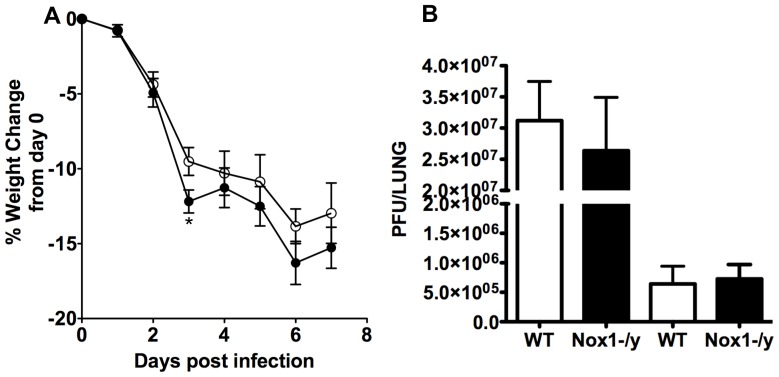
Effect of HkX-31 influenza A virus infection on viral titer and body weight in WT and Nox1^−/y^ mice. Mice were treated with 1×10^4^ PFU of HkX-31 (H3N2) strain of influenza A virus and (A) body weight recorded for up to 7 days post infection and (B) viral titer determined 3 and 7 days post infection. Data are shown as mean±SEM for 23 mice per group. *P<0.05 vs WT (ANOVA and Dunnett’s *post hoc* test).

Following HkX-31 infection, Nox1^−/y^ mice generally lost a greater percentage of their bodyweight than WT mice, however this was only significantly different at Day 3 (Figure 8). Also, we observed no significant differences in viral titers between the WT and Nox1^−/y^ mice (Figure 8) demonstrating that Nox1 does not influence either viral replication or mechanisms of viral clearance.

### Effect of Nox1 Deletion on the Magnitude and Function of T Lymphocyte Subsets

**Figure 9:**
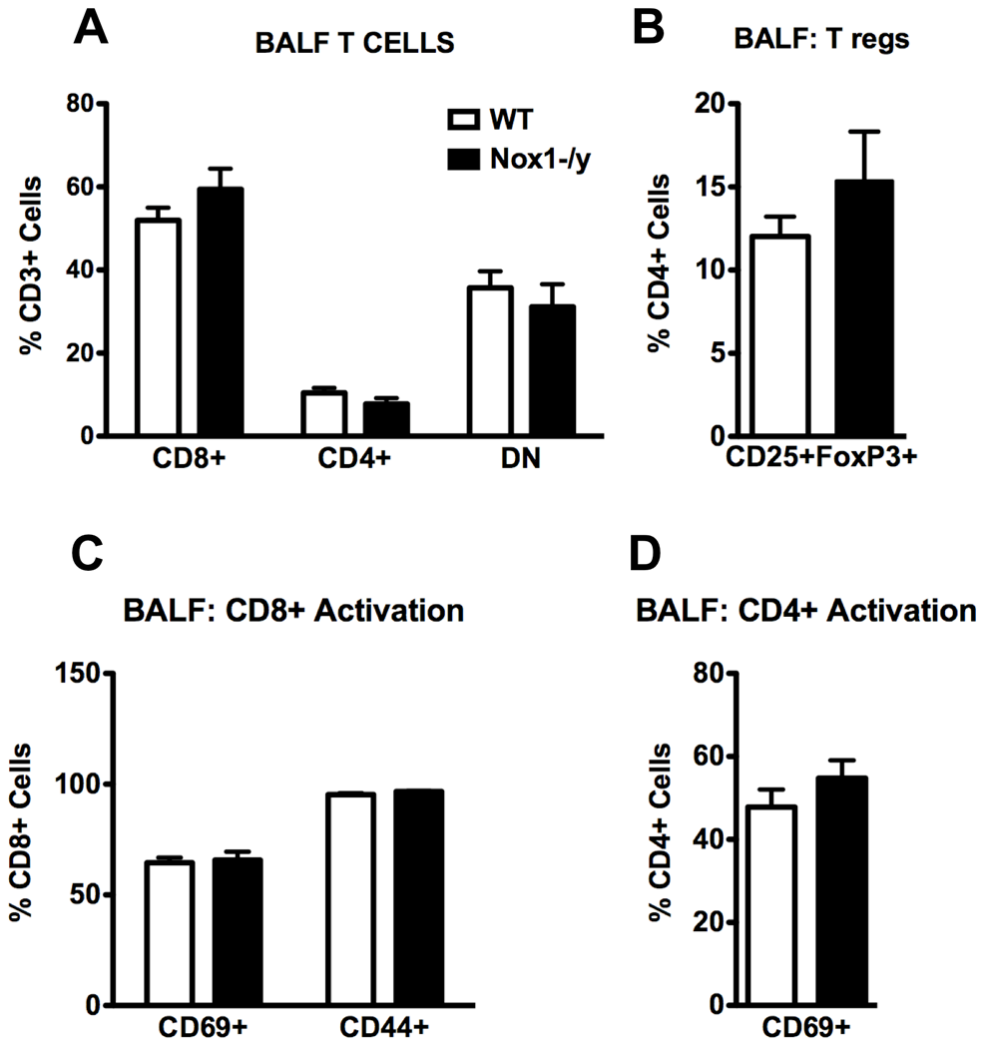
T cell enumeration from BALF obtained from HkX-31 influenza A virus infected WT and Nox1^−/y^ mice 7 days post infection. (A and B) Group data of flow cytometric plots for CD8^+^, CD4^+^ and CD4^+^CD25^+^FoxP3^+^ T cells from BALF from HkX-31 virus-infected WT and Nox1^−/y^ mice. Data are shown as mean ± SEM of 5 individual mice. (C and D) Group data as mean ± SEM of 5 individual mice of the number of active i.e. CD69^+^ and CD44^+^ CD8^+^ and CD4^+^ T cell populations in the BALF.

We investigated the effect of Nox1^−/y^ deficiency on the number and functionality of CD8^+^ and CD4^+^ T cells and of T regulatory cells at Day 7 post infection. HkX-31 infection elicited T cell infiltration into the airways and there were no differences in the number of CD8^+^, CD4^+^ and Tregs in the BALF infiltrate of WT and Nox1^−/y^ mice (Figure 9). In addition, there were no differences in the numbers of CD8^+^ T cells expressing either CD44 or CD69 (activation markers), or in the numbers of CD4^+^ T cells expressing CD69, between lungs from WT and Nox1^−/y^ mice (Figure 9).

## Discussion

The role of the Nox1 oxidase in the inflammation caused by influenza infection has not previously been determined. The major finding of the present study is that the Nox1 oxidase expressed in lung endothelial and epithelial cells plays a somewhat unexpected role in suppressing the early burst in pro-inflammatory cytokine expression and in the oxidative stress and inflammation in the lungs following influenza virus infection. This protective effect of Nox1 is surprising given that ROS have previously been implicated in promoting the lung injury and dysfunction to influenza virus infection. Indeed, excessive ROS production by the Nox2 oxidase was shown to contribute to the early lung injury and dysfunction caused by influenza [4], [6]–[8]. Hence, the current study highlights the complex role ROS play in modulating influenza disease progression.

In the present study, we demonstrate localization of Nox1 protein in lung epithelium, in particular the alveolar epithelium, as well as in vascular endothelium. These observations are consistent with the recent work of Carnesecchi *et al* 2009 who showed localization of Nox1 mRNA to the lung alveolar epithelial and endothelial cells [9]. This pattern of expression of Nox1 in the lung places the enzyme at primary sites to orchestrate the inflammation caused by an influenza infection. However, surprisingly the absence of Nox1 resulted in a marked increase in a number of pro-inflammatory cytokines that are key in regulating the inflammation to influenza viruses at an early stage of the infection i.e. Day 3. The suite of cytokines and chemokines assessed here, namely, TNF-α, IL-6, CCL2, CCL3, CXCL2 and IL-1β, are known to promote the inflammation caused by influenza at early stages of the infection phase, i.e. during the cytokine storm, and have been shown to underpin the pathology and morbidity [10], [11]. Indeed, early dysregulation of innate cellular and cytokine responses predict disease severity and death arising from highly pathogenic inﬂuenza virus infection [12]–[14] and are associated with symptoms in humans [15]. We also assessed cytokine and chemokine production at 7 days post infection. Surprisingly, some of the same pro-inflammatory cytokines and chemokines i.e. CCL2, CCL3, CXCl2 and GM-CSF that were significantly elevated in the lungs of Nox1^−/y^ mice at D3 were, by contrast, slightly but significantly lower at D7. However, at the same Day 7 time point the reduced expression of the anti-inflammatory cytokine IL-10 perhaps counteracts the reduction in pro-inflammatory cytokines. Overall it appears that at Day 7, there may be a balance reached between pro- and anti-inflammatory cytokines that help resolve the infection. Indeed at Day 7, there is a significant decrease in viral titer compared to Day 3 indicating resolution of infection. Overall, whether ROS stimulate or suppress cytokine and chemokine production is highly complex and we suggest will be dependent on a number of factors including the level of ROS generated, the type of ROS generated (i.e. superoxide, hydrogen peroxide etc.), the subcellular compartmentalization of ROS generation and suite of transcription factors expressed within the vicinity of ROS that ultimately regulate the expression of the cytokines and chemokines. These factors certainly warrant further investigation.

The present study also demonstrated that Nox1^−/y^ mice infected with HkX-31 influenza A virus displayed a modest but increased weight loss at Day 3 compared to WT mice, reflecting the exacerbated pro-inflammatory cytokine/chemokine expression profile in these mice at that time point. In addition, the lungs of Nox1^−/y^ mice displayed a significantly greater degree of inflammation, as assessed by H & E staining. This was characterized by an enhanced presence of inflammatory cells, particularly within the peri-bronchial and peri-vascular regions that persisted into D7 after the influenza insult. Interestingly the pattern of inflammation mirrored the localization pattern of Nox1, i.e. peri-vascular inflammation coinciding with Nox1 expression in endothelium, and the alveolitis coinciding with Nox1 expression in the alveolar epithelium. We also assessed BALF inflammation after a pulmonary challenge with Hk-X31 influenza A virus. At Day 3, lungs of infected Nox1^−/y^ mice contained slightly higher numbers of neutrophils in the BALF compared to WT lungs, but had fewer macrophages. The enhanced neutrophilia is possibly a consequence of an increase in expression of the neutrophil chemotactic chemokine CXCL2 and the cytokine IL-1β. The biological significance of these relatively modest effects on BALF cellularity is unknown, but even the small increase in neutrophilia could conceivably be a major contributor to oxidative stress given the high capacity of neutrophils to generate ROS via Nox2 oxidase.

Macrophages and neutrophils that either reside in the lung or have been recruited to the lung play crucial roles in regulating the host response against influenza A virus infections. However, whether macrophages and neutrophils have an overall protective effect or detrimental effect is controversial. For instance depletion of macrophages in pigs resulted in a significant increase in mortality and histopathological features compared to controls suggesting that macrophages are indispensable for controlling H1N1 influenza A virus ( [16]). Similarly antibody depletion of neutrophils in mice resulted in an increase in disease severity following influenza infection of mice, however, the beneficial effects of neutrophils appears to be dependent on the virulence of the particular strain examined ( [17]). By contrast, several studies suggest that an excessive host innate response characterized by a large infiltration of macrophages and neutrophils is an underlying *cause* of the lung pathology caused by influenza A virus. Activated macrophages and neutrophils produce large amounts of superoxide, and in excessive amounts superoxide is capable of inducing significant injury to surrounding tissue *via* its ability to give rise to a number of ROS and reactive nitrogen species with a high oxidising capacity, such as OONO^−^ and OH^.^ [18]. These molecules have detrimental biological effects such as nitration and oxidation of macromolecules, including proteins and DNA, leading to apoptosis *via* the caspase 3 pathway [18]. We have previously shown that the primary source of superoxide in lungs from influenza-infected mice is Nox2 oxidase – as BALF cells isolated from Nox2^−/y^ mice produced almost no superoxide either basally or following stimulation with PDB [6]. Moreover the lungs of Nox2^−/y^ mice produced substantially less peroxynitrite than those from WT mice, indicating that Nox2 is the major source of peroxynitrite induced by influenza A virus infection [6]. In the present study, deletion of Nox1 resulted in a substantial increase in: 1) the Nox2-dependent, oxidative burst capacity of BALF inflammatory cells, which are predominantly macrophages and neutrophils; and 2) oxidative stress (i.e. 3-nitrotyrosine staining) in a number of locations in the lungs. It is unlikely that the increase in Nox2 dependent oxidative burst and lung oxidative stress is due to a compensatory increase in Nox2 expression in the Nox1^−/y^ mice, as we have shown in the present study and as shown by Carnesecchi *et al* [9]. A possible reason for the enhanced oxidative burst by inflammatory cells is greater priming of Nox2 oxidase by pro-inflammatory mediators such as TNF-α, IL-1β and GM-CSF, all of which were elevated in naïve Nox1^−/y^ mice lungs compared to WT resulting in an increase in activation of Nox2 oxidase. Elevated degrees of oxidative stress were observed in the lung epithelium, vascular endothelium, and peri-bronchial and peri-vascular regions. This pattern of oxidative stress is most likely due to the enhanced presence of inflammatory cells observed in these regions. Another potential cause of the increased oxidative stress seen in Nox1^−/y^ lungs is reduced expression of the transcription factor Nrf2, which plays an important role in transcriptional upregulation of several antioxidant genes since previous studies have shown that Nrf2 expression is under the influence of Nox1 oxidase [19]. This could lead to a reduction in the antioxidant profile in epithelium resulting in the enhanced proinflammatory cytokine release. As mentioned above the increased cytokine levels in the lung milieu could activate/prime Nox2 in phagocytes resulting in the enhanced oxidative stress. These potential mechanisms of crosstalk between Nox1 and Nox2 oxidases are speculative at this stage but certainly warrant further investigation.

We also addressed whether Nox1 deletion influences the adaptive immune response that is ultimately responsible for clearing influenza viruses from the lungs. To achieve this we evaluated the amounts of several subsets of T lymphocytes including cytotoxic CD8^+^ T lymphocytes, effector CD4^+^ T cells and regulatory T lymphocytes i.e. CD25^+^CD4^+^FoxP3^+^. In addition we assessed the activation state of the CD8^+^ and CD4^+^ T cells by measuring expression of the cell surface activation markers CD69 and CD44. Our findings indicate that Nox1 does not influence T cell-mediated immunity, as there were no differences in the numbers of these relevant T lymphocyte subsets between WT and Nox1^−/y^ mice before or after influenza infection. As further evidence of an intact adaptive immune response, viral titers were similar at both D3 and D7 between WT and Nox1^−/y^ mice lungs. Overall, these findings indicate that Nox1 is a strong suppressor of airways and lung inflammation and oxidative stress that occurs during the early stages of the infection phase, particularly when the innate immune system is maximally active. By contrast, Nox1 appears to have a minor role in regulating the adaptive arm of the immune response that clear the virus.

Our findings that Nox1 and Nox2 oxidases have profoundly different effects in regulating the inflammation and oxidative stress caused by influenza A virus infection warrants further investigation. Indeed at face value, it is intriguing that these enzymes possess such contrasting effects, given that they generate the same enzymatic product i.e. superoxide [20], [21]. A possible reason for this opposition is related to differences in cell type and oxidase properties. Specifically, Nox2 is highly expressed in phagocytic cells and generates high levels of extracellular ROS to kill invading pathogens with a side-effect of this action being the production of peroxynitrite leading to oxidative damage to surrounding host tissues. By contrast, as shown in the present study Nox1 is expressed in airway epithelial cells and as opposed to Nox2 is responsible for low-level intracellular ROS production for cell signalling purposes [22] [23].

In conclusion, the present study evaluated the role of Nox1 oxidase in the lung inflammation and oxidative stress caused by influenza A virus infection. It demonstrates for the first time a protective effect of Nox1 following infection by an external pathogen, in this case influenza viruses. It remains to be determined whether Nox1 has similar protective effects against the lung inflammation and injury evoked by other viruses such as rhinovirus and respiratory syncytial virus (RSV), or by acute or chronic bacterial and fungal infection. Our study also highlights intriguing differences between Nox1 and Nox2 oxidases in regulation of lung inflammation and injury following influenza A virus infection. Surprisingly, the overall effect of Nox1 appears to be anti-inflammatory and to protect the host from oxidative stress. By contrast, Nox2 promotes inflammation, and is responsible for the oxidative stress-dependent lung injury and dysfunction. Our findings might explain why ROS scavengers generally fail to provide significant protection against influenza because they are likely to remove both the detrimental toxic effects of Nox2-derived ROS and the protective properties of Nox1-derived ROS. Therefore, selective targeting of Nox2 oxidase is likely to be a better therapeutic strategy than utilizing non-selective ROS scavengers. When used in combination with anti-viral drugs, selective Nox2 inhibitors might be an effective approach to target the host immune response for influenza A virus therapy.

## Materials and Methods

### Mice

Male wild type (WT, C57BL/6) and Nox1 deficient (Nox1^−/y^) mice (C57BL/6 background) aged between 7 and 12 weeks and weighing ∼25 g were bred at the Department of Pharmacology at Monash University. The animals were housed under specific pathogen-free conditions at 20°C on a 12 h day/night cycle in sterile micro-isolators and fed a standard sterile diet of Purina mouse chow with water allowed *ad libitum*.

### Animal Ethics Statement

The experiments described in this manuscript were approved by the Animal Experimentation Ethics Committee of The University of Melbourne and conducted in compliance with the guidelines of the National Health and Medical Research Council (NHMRC) of Australia on animal experimentation.

### Infection of WT and Nox1^−/y^ Mice

Male naïve WT and Nox1^−/y^ mice were anaesthetized by methoxyflurane inhalation and infected with 1×10^4^ plaque forming units (PFU) of HkX-31 strain (H3N2) *i.n.* in 35 µl of PBS. At days 3 (peak of viral titres) or 7 (resolution of infection) following influenza infection, mice were euthanized by an *i.p.* injection of sodium pentobarbitone (360 mg/kg).

### Quantitative Real-Time PCR

Whole lungs were perfused free of blood via right ventricular perfusion with 10 ml of pre-warmed saline, rapidly excised en bloc, blotted and snap frozen in liquid nitrogen. Total RNA was extracted from whole lung tissue (15 mg) using the RNeasy mini kits (Qiagen). RNA was transcribed into cDNA with high capacity RNA to cDNA kit (Applied Biosystems) for use in real time PCR reactions with Applied Biosystems pre-developed assay reagents and 18S rRNA internal control as previously described [24]–[27].

### Histology

Histology was performed as previously described [6], [24], [25], [28], [29] and sections stained with haematoxylin and eosin (H&E) for general histopathology. Airway inflammation of H&E-stained lung sections was evaluated on a subjective scale of 0, 1, 2, 3, 4, or 5 (corresponding to no inflammation and very mild, mild, moderate, marked, and severe inflammation, respectively) on randomized, blinded sections by two independent readers as previously published [28], [29]. Tissues were graded for peribronchial inflammation and alveolitis in multiple random fields per section [28], [29].

### Airways Inflammation and Differential Cell Counting

Lungs from each mouse were lavaged *in situ* with a 400 µl volume, followed by three 300 µl volumes of PBS. The total number of viable cells in the BALF was determined, cytospins prepared using 50 to 200 µl BALF, and cells differentiated by standard morphologic criteria as previously described [6]. The remaining BALF was spun at 800 g for 5 min to pellet cells for flow cytometry and superoxide detection.

### Superoxide Detection with L-O12 Enhanced Chemiluminescence

BALF inflammatory cells were exposed to the chemiluminescent probe, L-O12 (100 µM; Wako laboratories, Japan) in the absence (for basal measurements) or presence of the PKC and NADPH oxidase activator, phorbol dibutyrate (1 µM; Sigma) and dispensed into 96 well white opti-plates for luminescence reading with the TopCount (Perkin Elmer Packard). Photon emission was recorded from each well every 2 min and averaged over 45 min. Individual data points for each group were derived from the average of 3 replicates. We have previously shown incubation of cells with superoxide dismutase (SOD; 600 U/ml) to inactivate superoxide almost abolishes the chemiluminescence signal verifying that the L-O12 chemiluminescence signal was due to superoxide [6].

### Western Blotting

Western blotting was performed as we have previously shown [30]–[34]. Lungs were snap-frozen in liquid nitrogen and pulverised using a mortar and pestle. Following pulverisation, 0.4–0.5 ml of Laemmli buffer (5% glycerin, 2.5% mercaptoethanol, 1.5% sodium dodecyl sulphate (SDS), 50 mmol/L Tris-HCl, pH 8, and 0.05 mg/mL bromphenol blue) was added to each sample. Tissue homogenates were then sonicated (cycle 0.5, amplitude 80%) for 10 s using a Hielscher ultrasonic processor (UPSOH, Germany) and then heated on a heating block at 37°C for 10 min. The supernatant was removed by centrifugation at 15,000×g for 5 min at 4°C, and samples were stored at –20°C until analysis. Protein concentrations of tissue samples were determined using the RC DC™ protein kit according to the manufacturer’s specifications. Matched protein amounts of varying concentration (depending on the tissue type) were loaded onto 10% polyacrylamide gels. Samples were then resolved by SDS–polyacrylamide gel electrophoresis and transferred onto a polyvinylidene fluoride (PVDF) membrane. Following transfer, membranes were blocked in 5% non-fat dry milk (Bio-Rad blotting-grade blocker) for 1 h at room temperature and then incubated overnight at 4°C with a Nox2 antibody, (monoclonal; BD Transduction Laboratories; dilution 1∶1000). Membranes were then washed three times at 10 min intervals with Tris-buffered saline-tween (TBS-T) and then incubated for 1 h at room temperature with anti-mouse secondary antibody (Jackson ImmunoResearch; dilution 1∶10000) conjugated to horseradish peroxidase. Membranes were again washed in TBS-T (3×10 min) and immunoreactive bands were visualized following 1–30 min exposure to enhanced chemiluminescence (ECL) reagent (Blok-CH, Millipore) or (GE Healthcare ECL-advance) on X-ray film (Super RX; Kodak). Immunoreactive bands were quantified using ChemiDoc XRS Imager and Quantity One software (Bio-Rad). Following visualization, membranes were stripped in 0.5 M NaOH for 20 min and incubated with a primary antibody against β-actin (monoclonal; Sigma, St Louis, MO, USA; dilution 1∶5000) to assess protein loading and for normalization of immunoreactive bands.

### Immunohistochemistry for 3-NT

Lung sections from WT and Nox1^−/y^ mice infected with Hk-X31 were deparaffinised, fixed in acetone for 15 min and then washed in 0.01 M phosphate buffered saline (PBS, pH 7.4; 3×10 min) before staining for 3-NT, as previously published [6]. Tissue mounted sections were incubated in either mouse monoclonal anti-3-nitrotyrosine (1∶50, AbCAM), mouse monoclonal anti-Nox2 (1∶250, AbCAM), mouse monoclonal anti-Nox1 (1∶250; AbCAM) overnight in a humid box. The following day, tissues were washed in 0.01 M PBS (3×10 min) to remove any excess antibody, and incubated in a biotinylated anti-mouse IgG reagent for 10 min for 3-nitrotyrosine studies or a goat anti-rabbit Alexa fluor 488 (Invitrogen; 1∶500) secondary antibody for Nox1. Lung sections were then washed in 0.01 M PBS (3×10 min) and Fluorescein Avidin DCS (Vector Laboratories) was applied for 5 min. Sections were washed in 0.01 M PBS (3×10 min) and cover slipped. Slides were viewed and photographed on an Olympus fluorescence microscope. Several independent researchers were blinded throughout the experiment and all the appropriate primary and secondary controls were performed.

### Viral Titres

Lungs from influenza virus-infected mice were removed, weighed and homogenised in 2 ml of RPMI medium 1640. The plaque assay on Madin-Darby Canine Kidney (MDCK) cell monolayers as previously published [35] was used to quantify viral titers.

### Flow Cytometry

BALF leukocytes isolated from WT and Nox1^−/y^ mice were centrifuged (800×g), washed twice with PBS and containing 0.5% BSA (staining buffer), and 1×10^6^ cells re-suspended in staining buffer. The cells were then stained for 25 minutes at 4°C with fluorescently labeled antibodies for surface markers including CD4, CD8, CD69, CD44 and CD25. All surface antibodies were obtained from BD Biosciences. Following surface staining, cells were washed twice with staining buffer and then fixed and permeabilized using a FoxP3/T regulatory cell kit (eBioscience) according to manufacturer’s instructions. Intracellular staining for the T regulatory cell transcription factor FoxP3 was performed on fixed/permeabilized cells through incubation with a fluorescently labeled anti-FoxP3 antibody (eBioscience) at room temperature for 15 minutes. Cells were then washed twice with staining buffer and re-suspended in staining buffer containing 1% paraformaldehyde until analyzed using LSR-II flow cytometer with DIVA software (BD Bioscience). Data was analyzed with Flowjo software (Version 9.3.3, Treestar).

### Materials

Antibodies for staining were used in different multi-color combinations and were either from BD Biosciences (V450 anti-CD45 (30-F11); V500 anti-CD3 (145-2C11); APC-Cy7 anti-CD44 (IM7); PE anti-CD69 (H1.2F3); PE-Cy7 anti-CD8a (53-6.7); APC anti-CD25 (7D4)) or eBioscience (FITC anti-CD4 (RM4-5), PE anti-FoxP3 (FJK-16s)). An eBioscience Mouse Regulatory T Cell staining kit (88-8111) was used to stain T regulatory cells from BALF.

### Statistical Analyses

Data are presented as mean ± standard error of the mean (SEM); *n* represents the number of mice. Differences in BALF cell types and whole lung mRNA expression were determined by analysis of variance (ANOVA) followed by Dunnett *post hoc* test for multiple comparisons, where appropriate. In some cases, Student’s unpaired *t*-test was used to determine if there were significant differences between means of pairs. All statistical analyses were performed using GraphPad Prism for Windows (Version 5.0). In all cases, probability levels less than 0.05 (*P<0.05) were taken to indicate statistical significance.

## Supporting Information

Figure S1 **Lung cytokine and chemokine levels.** Effect of HkX-31 (H3N2) influenza A virus infection on CCL2, CCL3, CXCL2, IL-1β, IL-6, TNF-α, IFN-γ, GMCSF and IL-10 mRNA expression in whole lung obtained from naïve WT and Nox1^−/y^ mice. Gene expression levels are shown as fold change relative to naïve (uninfected) WT mice after normalisation to 18S rRNA (housekeeping gene). Data are shown as mean ± SEM of 4 individual mice. *P<0.05 vs WT (Students’ unpaired t test).
(TIF)

Figure S2 **Lung histology showing representative perivascular inflammation in response to HkX-31** **influenza A virus infection in mice.** Hematoxylin and eosin stained paraffin sections of lungs from WT and Nox1^−/y^ mice obtained at Day 3. Note, magnification of X400.
(TIF)

Figure S3 **Effect of Nox1 deletion on Nox2 expression in naïve and HkX-31 influenza A virus infected mice.** Western blot image showing protein expression of Nox2 protein in lung tissue taken from naïve wild type and Nox1^−/y^ mice (lanes 1 and 2), WT and Nox1^−/y^ lungs at D3 (lanes 3 and 4) and at Day 7 (lanes 5 and 6). This is a representative blot from 4 separate experiments.
(TIF)

Figure S4 **Effect of HkX-31 influenza A virus infection on lung oxidative stress (i.e. peroxynitrite generation).** Representative sections of lung tissue obtained from WT and Nox1^−/y^ mice infected with HkX-31 were incubated with mouse monoclonal anti-3-nitrotyrosine antibody (1∶50) followed by biotinylated anti-mouse IgG reagent.
(TIF)

Figure S5 **Effect of Nox2 deletion on HkX-31 influenza A virus infection induced lung oxidative stress (i.e. peroxynitrite generation).** Representative sections of lung tissue obtained from Nox2^−/y^ mice infected with HkX-31 were incubated with mouse monoclonal anti-3-nitrotyrosine antibody (1∶50) followed by biotinylated anti-mouse IgG reagent.
(TIF)

## References

[1] Doherty PC, Turner SJ, Webby RG, Thomas PG (2006) Influenza and the challenge for immunology. Nat Immunol 7: 449–455. 16622432 10.1038/ni1343

[2] Newall AT, Scuffham PA, Hodgkinson B (2007) Economic Report into the cost of influenza to the Australian Health System; Report to the Influenza Specialist Group. 1–19.

[3] Akaike T, Noguchi Y, Ijiri S, Setoguchi K, Suga M, et al (1996) Pathogenesis of influenza virus-induced pneumonia: involvement of both nitric oxide and oxygen radicals. Proc Natl Acad Sci U S A 93: 2448–2453. 8637894 10.1073/pnas.93.6.2448 PMC39817

[4] Imai Y, Kuba K, Neely GG, Yaghubian-Malhami R, Perkmann T, et al (2008) Identification of oxidative stress and Toll-like receptor 4 signaling as a key pathway of acute lung injury. Cell 133: 235–249. 18423196 10.1016/j.cell.2008.02.043 PMC7112336

[5] Oda T, Akaike T, Hamamoto T, Suzuki F, Hirano T, et al (1989) Oxygen radicals in influenza-induced pathogenesis and treatment with pyran polymer-conjugated SOD. Science 244: 974–976. 2543070 10.1126/science.2543070

[6] Vlahos R, Stambas J, Bozinovski S, Broughton BR, Drummond GR, et al (2011) Inhibition of Nox2 oxidase activity ameliorates influenza A virus-induced lung inflammation. PLoS Pathog 7: e1001271. 21304882 10.1371/journal.ppat.1001271 PMC3033375

[7] Vlahos R, Stambas J, Selemidis S (2012) Suppressing production of reactive oxygen species (ROS) for influenza A virus therapy. Trends Pharmacol Sci 33: 3–8. 21962460 10.1016/j.tips.2011.09.001

[8] Snelgrove RJ, Edwards L, Rae AJ, Hussell T (2006) An absence of reactive oxygen species improves the resolution of lung influenza infection. Eur J Immunol 36: 1364–1373. 16703568 10.1002/eji.200635977

[9] Carnesecchi S, Deffert C, Pagano A, Garrido-Urbani S, Metrailler-Ruchonnet I, et al (2009) NADPH oxidase-1 plays a crucial role in hyperoxia-induced acute lung injury in mice. Am J Respir Crit Care Med 180: 972–981. 19661248 10.1164/rccm.200902-0296OC PMC2778156

[10] La Gruta NL, Kedzierska K, Stambas J, Doherty PC (2007) A question of self-preservation: immunopathology in influenza virus infection. Immunol Cell Biol 85: 85–92. 17213831 10.1038/sj.icb.7100026

[11] Teijaro JR, Walsh KB, Cahalan S, Fremgen DM, Roberts E, et al (2011) Endothelial cells are central orchestrators of cytokine amplification during influenza virus infection. Cell 146: 980–991. 21925319 10.1016/j.cell.2011.08.015 PMC3176439

[12] Bermejo-Martin JF, Ortiz de Lejarazu R, Pumarola T, Rello J, Almansa R, et al (2009) Th1 and Th17 hypercytokinemia as early host response signature in severe pandemic influenza. Crit Care 13: R201. 20003352 10.1186/cc8208 PMC2811892

[13] Kobasa D, Takada A, Shinya K, Hatta M, Halfmann P, et al (2004) Enhanced virulence of influenza A viruses with the haemagglutinin of the 1918 pandemic virus. Nature 431: 703–707. 15470432 10.1038/nature02951

[14] Kobasa D, Jones SM, Shinya K, Kash JC, Copps J, et al (2007) Aberrant innate immune response in lethal infection of macaques with the 1918 influenza virus. Nature 445: 319–323. 17230189 10.1038/nature05495

[15] Kaiser L, Fritz RS, Straus SE, Gubareva L, Hayden FG (2001) Symptom pathogenesis during acute influenza: interleukin-6 and other cytokine responses. J Med Virol 64: 262–268. 11424113 10.1002/jmv.1045

[16] Kim HM, Lee YW, Lee KJ, Kim HS, Cho SW, et al (2008) Alveolar macrophages are indispensable for controlling influenza viruses in lungs of pigs. J Virol 82: 4265–4274. 18287245 10.1128/JVI.02602-07 PMC2293066

[17] Tate MD, Ioannidis LJ, Croker B, Brown LE, Brooks AG, et al (2011) The role of neutrophils during mild and severe influenza virus infections of mice. PLoS One 6: e17618. 21423798 10.1371/journal.pone.0017618 PMC3056712

[18] Szabo C, Ischiropoulos H, Radi R (2007) Peroxynitrite: biochemistry, pathophysiology and development of therapeutics. Nat Rev Drug Discov 6: 662–680. 17667957 10.1038/nrd2222

[19] Malec V, Gottschald OR, Li S, Rose F, Seeger W, et al (2011) HIF-1 alpha signaling is augmented during intermittent hypoxia by induction of the Nrf2 pathway in NOX1-expressing adenocarcinoma A549 cells. Free Radic Biol Med 48: 1626–1635. 10.1016/j.freeradbiomed.2010.03.008 20347035

[20] Drummond GR, Selemidis S, Griendling KK, Sobey CG (2011) Combating oxidative stress in vascular disease: NADPH oxidases as therapeutic targets. Nat Rev Drug Discov 10: 453–471. 21629295 10.1038/nrd3403 PMC3361719

[21] Selemidis S, Sobey CG, Wingler K, Schmidt HH, Drummond GR (2008) NADPH oxidases in the vasculature: molecular features, roles in disease and pharmacological inhibition. Pharmacol Ther 120: 254–291. 18804121 10.1016/j.pharmthera.2008.08.005

[22] Oakley FD, Abbott D, Li Q, Engelhardt JF (2009) Signaling components of redox active endosomes: the redoxosomes. Antioxid Redox Signal 11: 1313–1333. 19072143 10.1089/ars.2008.2363 PMC2842130

[23] Miller FJ Jr, Chu X, Stanic B, Tian X, Sharma RV, et al (2010) A differential role for endocytosis in receptor-mediated activation of Nox1. Antioxid Redox Signal 12: 583–593. 19737091 10.1089/ars.2009.2857 PMC2861543

[24] Vlahos R, Bozinovski S, Chan SP, Ivanov S, Linden A, et al (2010) Neutralizing granulocyte/macrophage colony-stimulating factor inhibits cigarette smoke-induced lung inflammation. Am J Respir Crit Care Med 182: 34–40. 20203243 10.1164/rccm.200912-1794OC

[25] Vlahos R, Bozinovski S, Jones JE, Powell J, Gras J, et al (2006) Differential protease, innate immunity, and NF-kappaB induction profiles during lung inflammation induced by subchronic cigarette smoke exposure in mice. Am J Physiol Lung Cell Mol Physiol 290: L931–945. 16361358 10.1152/ajplung.00201.2005

[26] Chen H, Hansen MJ, Jones JE, Vlahos R, Anderson GP, et al (2007) Detrimental metabolic effects of combining long-term cigarette smoke exposure and high-fat diet in mice. Am J Physiol Endocrinol Metab 293: E1564–1571. 17940214 10.1152/ajpendo.00442.2007

[27] Prause O, Bossios A, Silverpil E, Ivanov S, Bozinovski S, et al (2009) IL-17-producing T lymphocytes in lung tissue and in the bronchoalveolar space after exposure to endotoxin from Escherichia coli in vivo–effects of anti-inflammatory pharmacotherapy. Pulm Pharmacol Ther 22: 199–207. 19121406 10.1016/j.pupt.2008.12.005

[28] Tate MD, Deng YM, Jones JE, Anderson GP, Brooks AG, et al (2009) Neutrophils ameliorate lung injury and the development of severe disease during influenza infection. J Immunol 183: 7441–7450. 19917678 10.4049/jimmunol.0902497

[29] Yatmaz S, Seow HJ, Gualano RC, Wong ZX, Stambas J, et al.. (2012) Glutathione Peroxidase-1 (GPx-1) Reduces Influenza A Virus-Induced Lung Inflammation. Am J Respir Cell Mol Biol. 10.1165/rcmb.2011-0345OC 23002098

[30] Harrison CB, Selemidis S, Guida E, King PT, Sobey CG, et al (2012) NOX2beta: A novel splice variant of NOX2 that regulates NADPH oxidase activity in macrophages. PLoS One 7: e48326. 23118986 10.1371/journal.pone.0048326 PMC3485160

[31] Harrison CB, Drummond GR, Sobey CG, Selemidis S (2010) Evidence that nitric oxide inhibits vascular inflammation and superoxide production via a p47phox-dependent mechanism in mice. Clin Exp Pharmacol Physiol 37: 429–434. 19843095 10.1111/j.1440-1681.2009.05317.x

[32] Judkins CP, Diep H, Broughton BR, Mast AE, Hooker EU, et al (2010) Direct evidence of a role for Nox2 in superoxide production, reduced nitric oxide bioavailability, and early atherosclerotic plaque formation in ApoE−/− mice. Am J Physiol Heart Circ Physiol 298: H24–32. 19837950 10.1152/ajpheart.00799.2009

[33] Peshavariya H, Dusting GJ, Jiang F, Halmos LR, Sobey CG, et al (2009) NADPH oxidase isoform selective regulation of endothelial cell proliferation and survival. Naunyn Schmiedebergs Arch Pharmacol 380: 193–204. 19337723 10.1007/s00210-009-0413-0

[34] Selemidis S, Dusting GJ, Peshavariya H, Kemp-Harper BK, Drummond GR (2007) Nitric oxide suppresses NADPH oxidase-dependent superoxide production by S-nitrosylation in human endothelial cells. Cardiovasc Res 75: 349–358. 17568572 10.1016/j.cardiores.2007.03.030

[35] Tannock GA, Paul JA, Barry RD (1984) Relative immunogenicity of the cold-adapted influenza virus A/Ann Arbor/6/60 (A/AA/6/60-ca), recombinants of A/AA/6/60-ca, and parental strains with similar surface antigens. Infect Immun 43: 457–462. 6693167 10.1128/iai.43.2.457-462.1984 PMC264316

