# Supplementary figures and images for: Nox1 Oxidase Suppresses Influenza A Virus-Induced Lung Inflammation and Oxidative Stress
Source: PLoS One. 2013 Apr 8;8(4):e60792. doi: 10.1371/journal.pone.0060792 (PMC3620107; doi:10.1371/journal.pone.0060792)

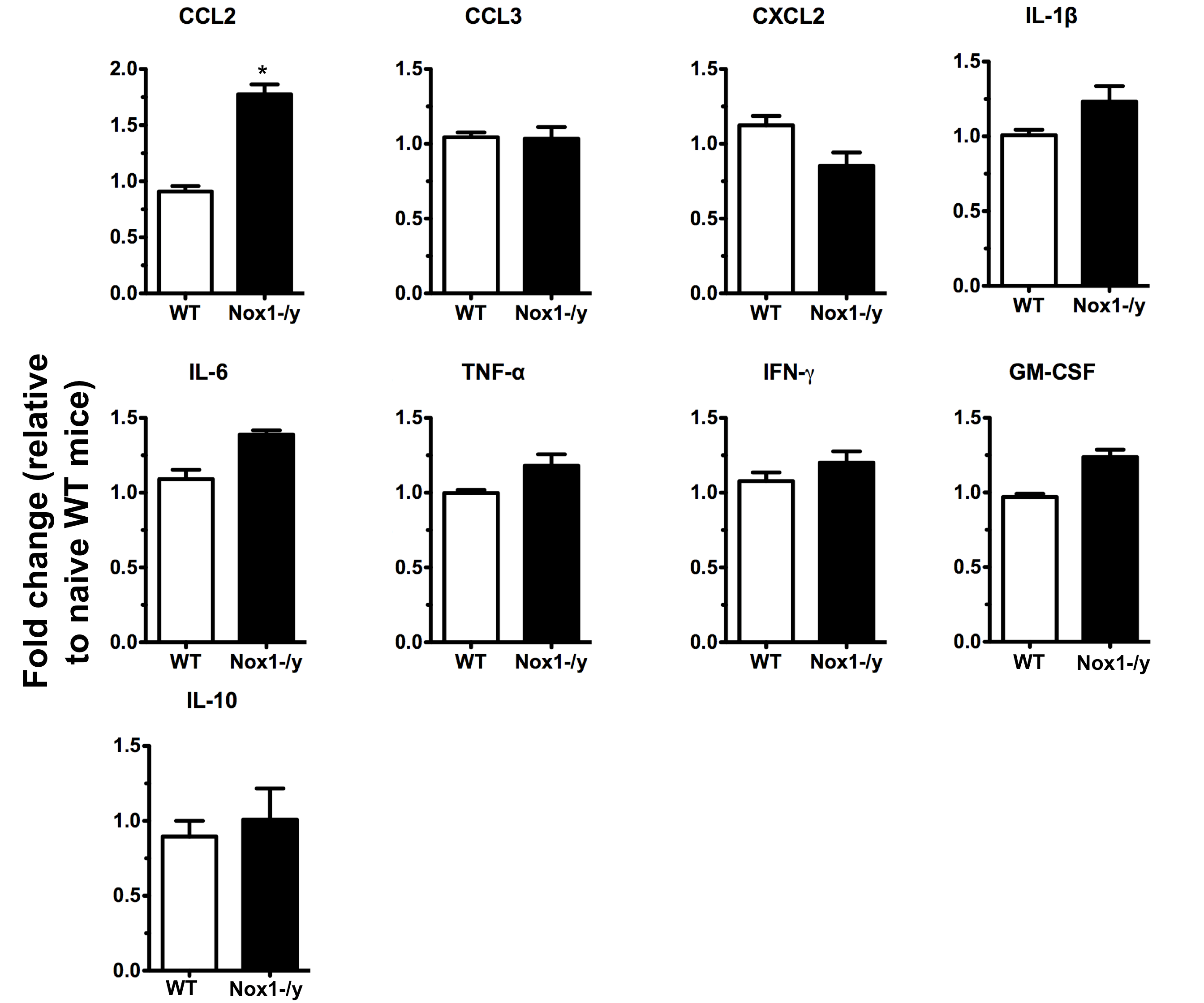

Supplement: Figure S1 — Lung cytokine and chemokine levels. Effect of HkX-31 (H3N2) influenza A virus infection on CCL2, CCL3, CXCL2, IL-1β, IL-6, TNF-α, IFN-γ, GMCSF and IL-10 mRNA expression in whole lung obtained from naïve WT and Nox1−/y mice. Gene expression levels are shown as fold change relative to naïve (uninfected) WT mice after normalisation to 18S rRNA (housekeeping gene). Data are shown as mean ± SEM of 4 individual mice. *P<0.05 vs WT (Students’ unpaired t test). (TIF) [file pone.0060792.s001.tif]

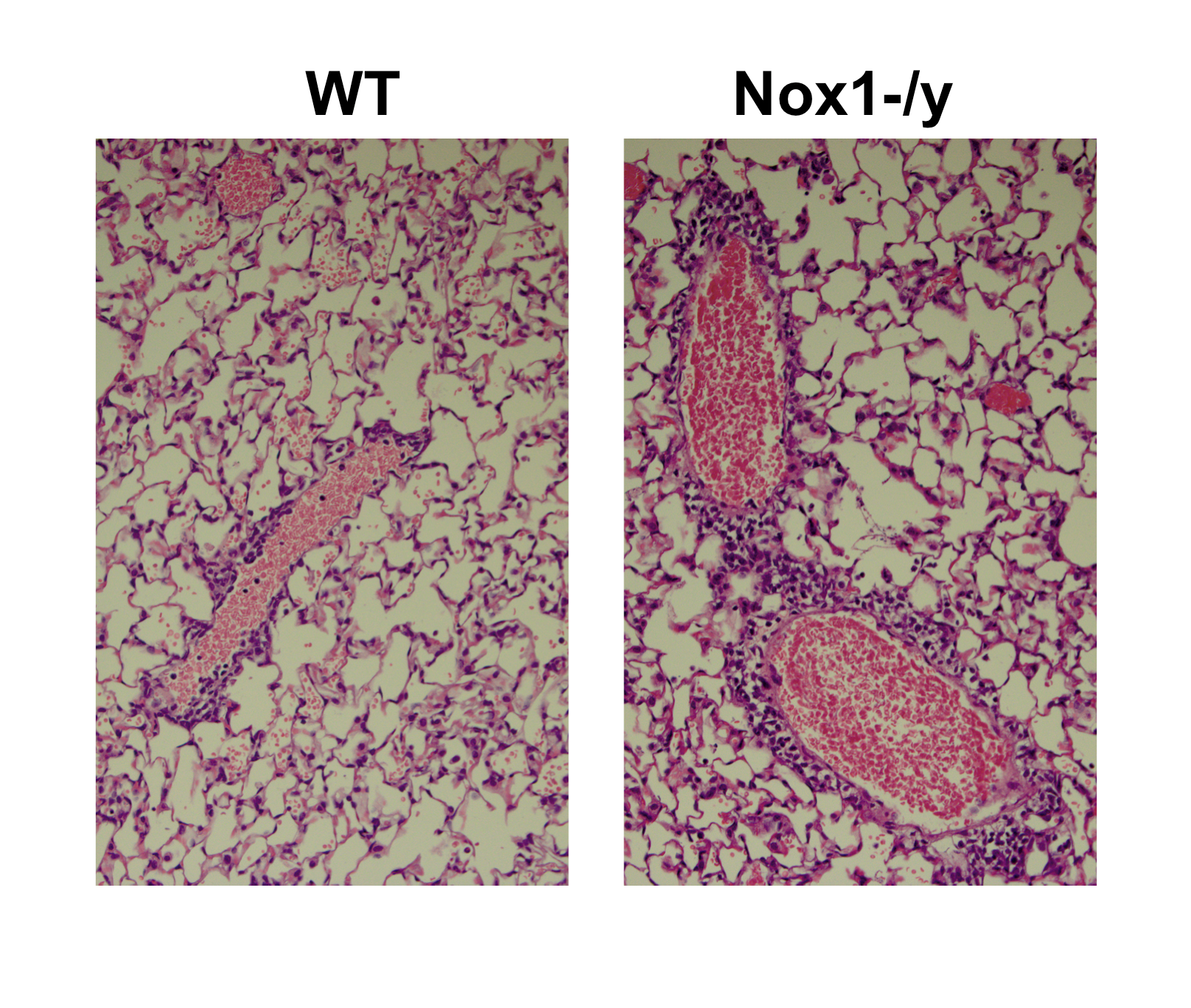

Supplement: Figure S2 — Lung histology showing representative perivascular inflammation in response to HkX-31 influenza A virus infection in mice. Hematoxylin and eosin stained paraffin sections of lungs from WT and Nox1−/y mice obtained at Day 3. Note, magnification of X400. (TIF) [file pone.0060792.s002.tif]

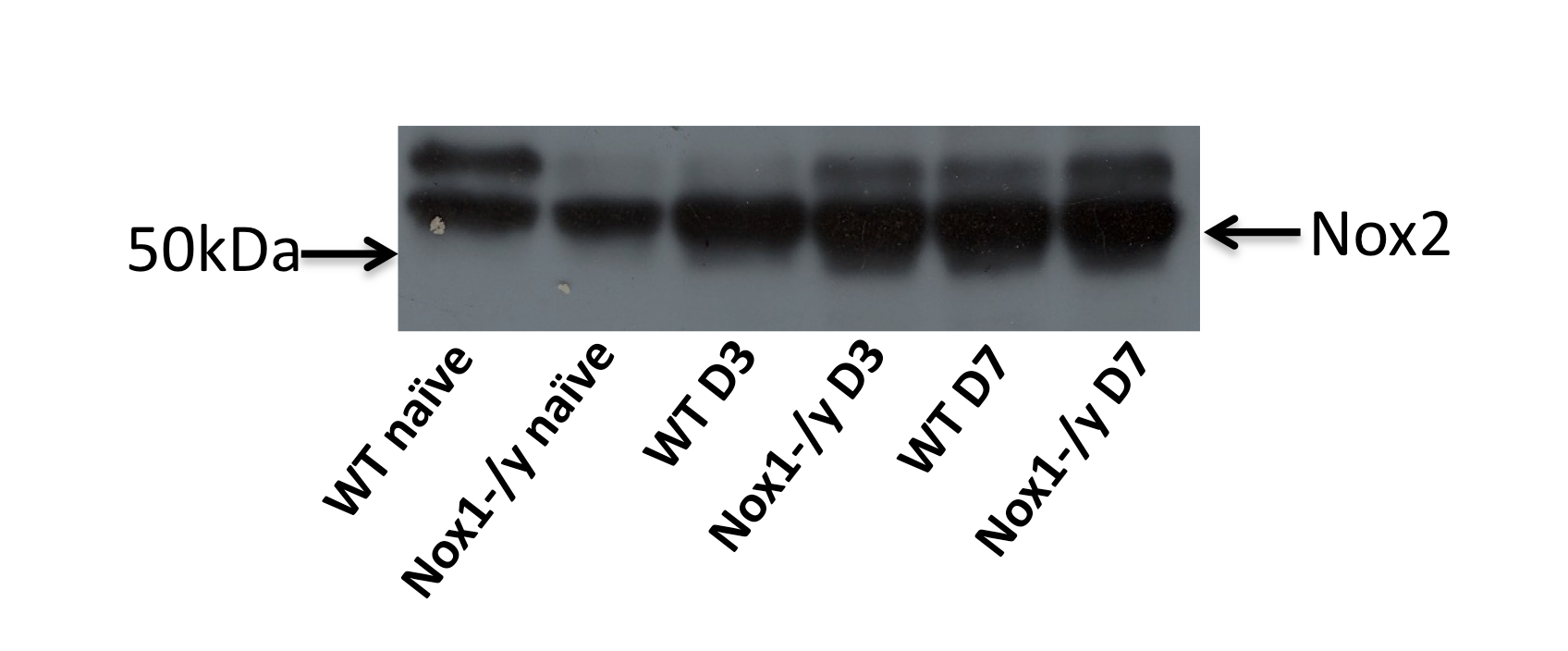

Supplement: Figure S3 — Effect of Nox1 deletion on Nox2 expression in naïve and HkX-31 influenza A virus infected mice. Western blot image showing protein expression of Nox2 protein in lung tissue taken from naïve wild type and Nox1−/y mice (lanes 1 and 2), WT and Nox1−/y lungs at D3 (lanes 3 and 4) and at Day 7 (lanes 5 and 6). This is a representative blot from 4 separate experiments. (TIF) [file pone.0060792.s003.tif]

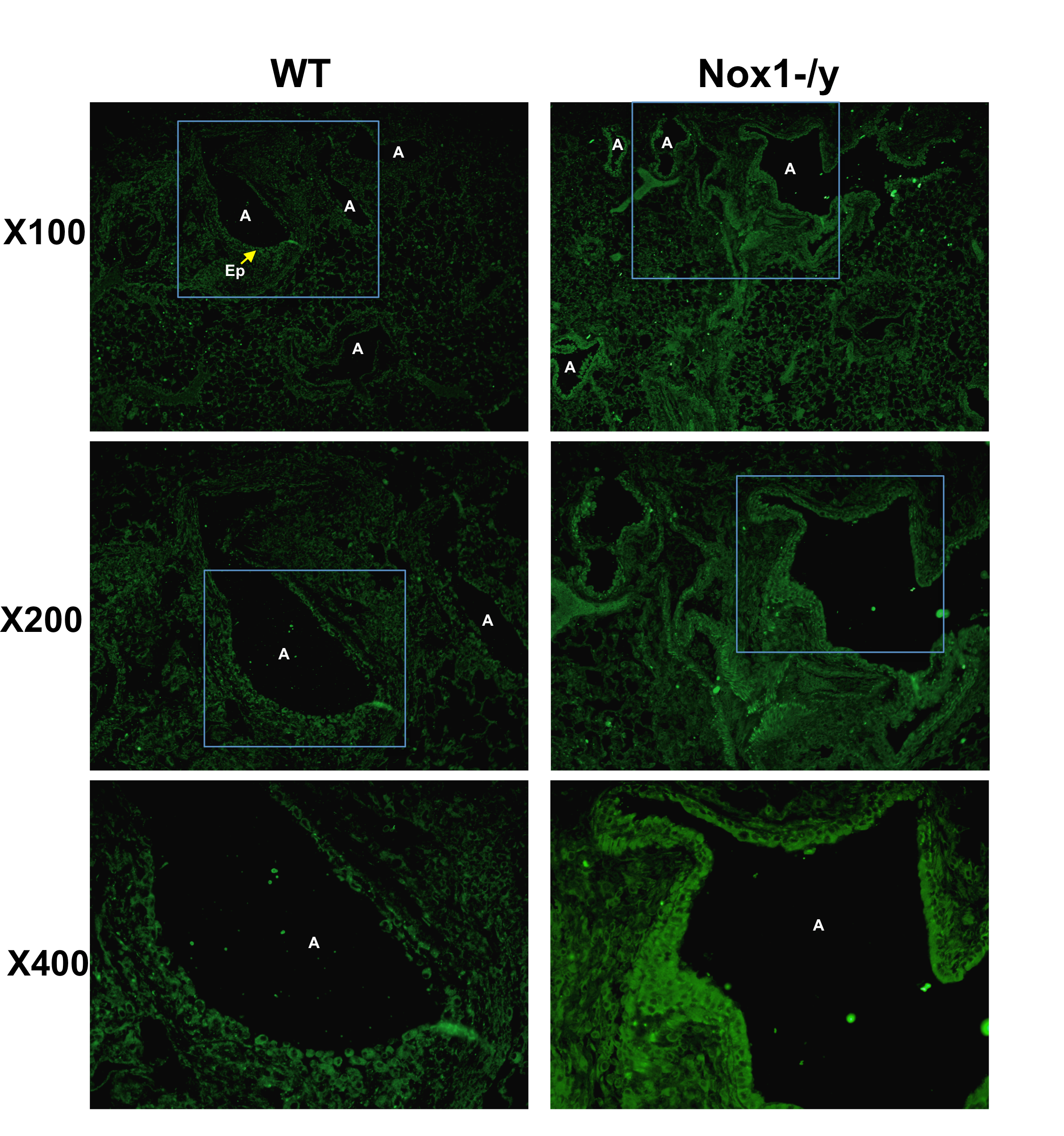

Supplement: Figure S4 — Effect of HkX-31 influenza A virus infection on lung oxidative stress (i.e. peroxynitrite generation). Representative sections of lung tissue obtained from WT and Nox1−/y mice infected with HkX-31 were incubated with mouse monoclonal anti-3-nitrotyrosine antibody (1∶50) followed by biotinylated anti-mouse IgG reagent. (TIF) [file pone.0060792.s004.tif]

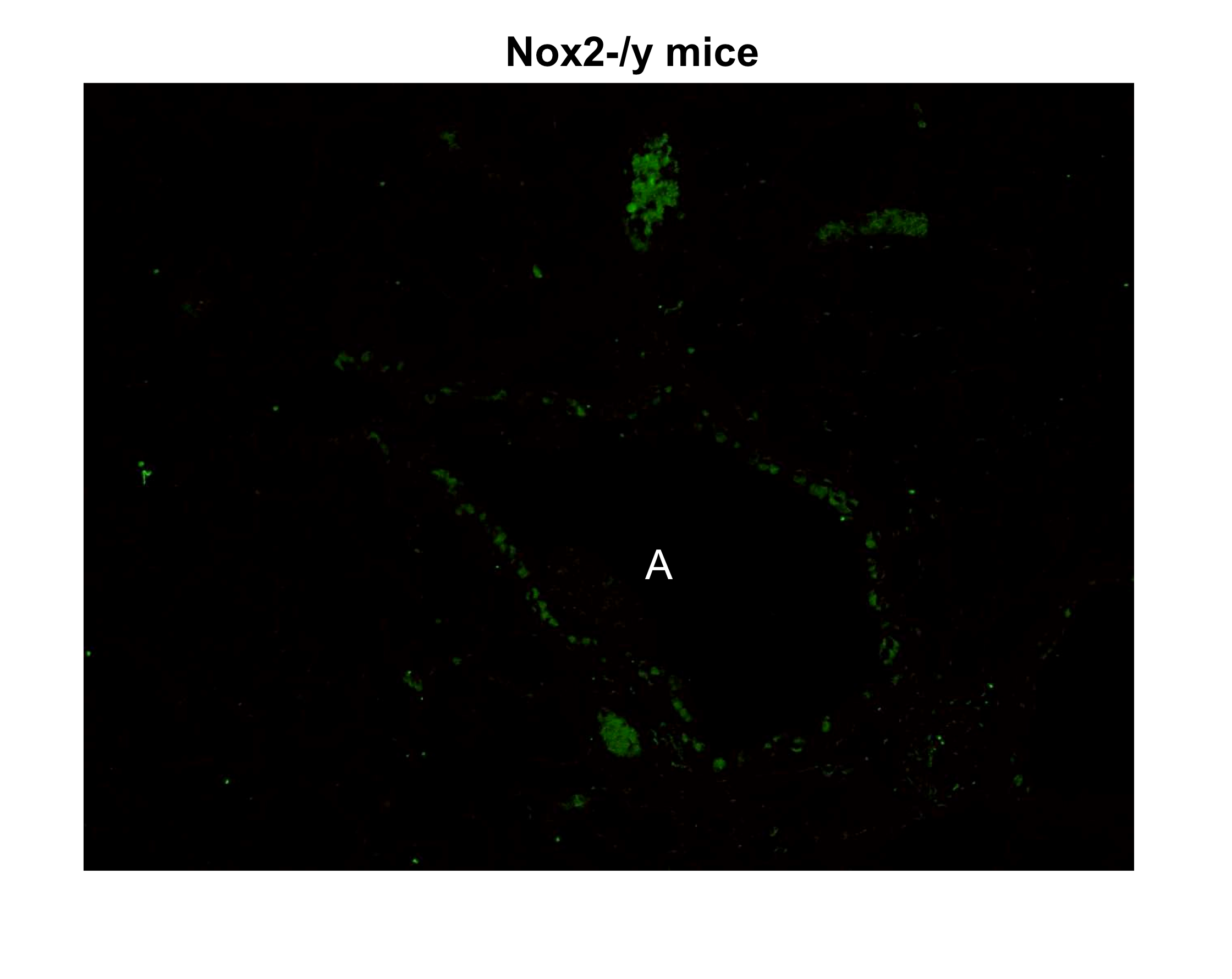

Supplement: Figure S5 — Effect of Nox2 deletion on HkX-31 influenza A virus infection induced lung oxidative stress (i.e. peroxynitrite generation). Representative sections of lung tissue obtained from Nox2−/y mice infected with HkX-31 were incubated with mouse monoclonal anti-3-nitrotyrosine antibody (1∶50) followed by biotinylated anti-mouse IgG reagent. (TIF) [file pone.0060792.s005.tif]
